# Dissipation of the Insecticide Cyantraniliprole and Its Metabolite IN-J9Z38 in Proso Millet during Cultivation

**DOI:** 10.1038/s41598-019-48206-0

**Published:** 2019-08-12

**Authors:** Jonghwa Lee, Min Woo Jung, Junghak Lee, Jiho Lee, Yongho Shin, Jeong-Han Kim

**Affiliations:** 1 Department of Agricultural Biotechnology and Research Institute of Agriculture and Life Sciences, Seoul National University, Seoul, 08826 Republic of Korea; 2 Present Address: Department of Veterinary and Animal Sciences, University of Massachusetts, Amherst, MA 01003 USA

**Keywords:** Environmental monitoring, Environmental monitoring, Environmental chemistry, Environmental chemistry

## Abstract

The dissipation patterns of cyantraniliprole and its metabolite IN-J9Z38 were investigated using proso millet (*Panicum miliaceum*) under open-field conditions to establish a pre-harvest interval. A simple and sensitive analytical method was developed for analyzing residues using ultra-high performance liquid chromatography coupled with tandem mass spectrometry (UHPLC-MS/MS) for multiple reaction monitoring of target compounds. The analytical method was validated in terms of the instrumental limit of quantitation, method limit of quantitation, linearity, accuracy, and precision. The method was successfully applied to the analysis of cyantraniliprole and IN-J9Z38 residues in the field samples of four plots, which were treated twice with an oil dispersion formulation, according to the date of pesticide treatment before harvest. In the case of cyantraniliprole in grain and straw, there was a 91.1 and 89.1% decrease, respectively, from the initial residues (14–7 days) to the final plot (40–30 days before harvest). However, IN-J9Z38 gradually increased over time, indicating that cyantraniliprole transformed into IN-J9Z38 during cultivation. The biological half-lives of total cyantraniliprole were 11.3 and 9.4 days for grain and straw, respectively. The results obtained in this study will inform regulation and management of pesticide use for the minor crop proso millet.

## Introduction

High-value agricultural products have a high margin of profit compared to their agricultural scale, and thus the lack of crop protection options has become a controversial issue in minor crop production^1^. While the definition of a minor crop varies from country to country, some of these definitions include: crops that are cultivated in a limited area, crops consumed in small amounts, or crops that result in less economic benefit than major crops. In Korea, the size of the cultivated area is used to classify a minor crop, with a minor crop defined as one that is cultivated in cultivation areas smaller than 1,000 hectares^2,3^. However, regardless of the cultivation scale or economic interest in a crop, appropriate use of pesticides is very important to protect crops against insects and diseases.

It is difficult to control insects and diseases when cultivating minor crops due to the limited number of pesticides registered for these target crops. In general, minor crops do not provide a big enough market for the pesticide manufacturer to rationalize the cost of registration testing or maintenance of pesticide registration. However, minor crops can play an important role in supplying diverse nutrients and foods^4^, as well as providing high economic value. Therefore, Korean government authorities including both the Korean Rural Development Administration and the Ministry of Food and Drug Safety have launched a program to register pesticides for minor crops through designed field dissipation studies after examining which pesticides can be used for certain minor crops.

Proso millet (*Panicum miliaceum*) is a widely cultivated millet belonging to a flowering plant in the family Poaceae, commonly known as Gramineae^5,6^. As a high grain-yielding crop, proso millet can be an important source of many nutrients to both humans and animals^7,8^. While proso millet is widely grown in East Asia, Russia, India, and Southern Europe^9^, it is still considered a minor crop in Korea along with sorghum and foxtail millet (known as Italian millet). This is because it has a smaller cultivation area and less productivity than other staple cereals such as rice, barley, and wheat. Since few pesticides have been registered for proso millet, registration of further pesticides is necessary for sustainable high-yielding production of proso millet.

Cyantraniliprole is a systemic diamide insecticide used specifically to control lepidopteran pests in agriculture^10^. It is a promising insecticide due to its unique mode of action in activating muscle ryanodine receptors, causing contraction and paralysis of some pests. In Korea, cyantraniliprole has already been registered for use against lepidopterous pests and aphids on several fruits and vegetables including apples, peaches, tomatoes, watermelons, and cucumbers^11^. Currently, no maximum residue level (MRL) has been set for a proso millet commodity in the CODEX list^12^. In Korea, there was no MRL of cyantraniliprole for proso millet before 2017, when this study was performed.

**Figure 1:**
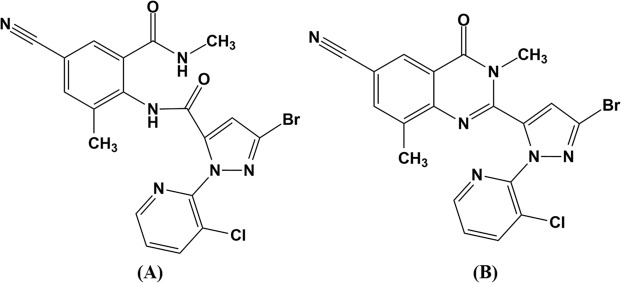
Chemical structure of cyantraniliprole (**A**) and IN-J9Z38 (**B**).

According to the international MRL of Codex Alimentarius, only the parent compound cyantraniliprole is included in the residue definition for compliance with the MRL in animal and plant commodities. However, for the purpose of estimating dietary intake for processed plant commodities, the sum of cyantraniliprole and IN-J9Z38 (Fig. 1) is defined as a cyantraniliprole residue. IN-J9Z38 is a metabolite generated by the ring closure of cyantraniliprole, and this compound is frequently formed as a result of environmental degradation or plant metabolism of cyantraniliprole^13,14^. In many studies, residual patterns of cyantraniliprole have been reported in soil, cucumbers, rice, tobacco, tomatoes, and watermelons^15–20^. However, to the best of our knowledge, there have been no studies on the residual behavior of cyantraniliprole in any millet species including proso millet.

The purpose of the study was: (a) to optimize and validate an analytical method for analyzing cyantraniliprole and IN-J9Z38 residues in proso millet (grain and straw) using an ultra-high performance liquid chromatography (UHPLC-MS/MS) combined with QuEChERS sample treatment, (b) to understand the residual pattern of cyantraniliprole in proso millet after spraying twice with a 10.26% oil dispersion (OD) formulation on an open-field system, and (c) to provide the necessary data for setting MRL and pre-harvest interval PHI for safe use of this pesticide during proso millet cultivation.

## Materials and Methods

### Chemicals and reagents

The analytical standard of cyantraniliprole (purity: 98%) was purchased from Wako Pure Chemical Industries (Osaka, Japan) and its metabolite IN-J9Z38 (purity: 97.3%) standard was supplied by FarmHanong company (Seoul, Republic of Korea). HPLC-grade acetonitrile was obtained from Fisher Scientific (Fair Lawn, NJ). QuEChERS extraction packages containing 4 g of magnesium sulfate, 1 g of sodium chloride, 1 g of trisodium citrate dehydrate, 0.5 g of disodium hydrogen citrate sesquihydrate, and dispersive solid phase extraction tubes containing 25 mg of primary secondary amine (PSA) and 150 mg of magnesium sulfate were purchased from Restek Corporation (Bellefonte, PA, USA). Ceramic homogenizer for QuEChERS extraction was obtained from Agilent Technologies (Santa Clara, CA, USA). Cyantraniliprole OD formulation with 10.26% active ingredient was purchased from the local agricultural market.

### Pesticide standard solution

Standard stock solutions of each pesticide at 100 μg/mL were prepared by dissolving analytical standards in 25 mL of acetonitrile. The mixtures of standard solution (10 μg/mL) were prepared by mixing 1 mL of each stock solution with acetonitrile to a total volume of 10 mL. Pesticide working solutions for instrumental analysis within the concentration range of 0.0025–0.25 μg/mL were prepared by serial dilution with acetonitrile. All of the stock solutions and working solutions were stored in amber glass vials at −20 °C until analysis.

### Field experiment

Proso millet was grown under open-field conditions in Hwaseong-si (Kyeonggi-do, Republic of Korea, 37°06′38.4′′N 126°54′44.1′′E). The proso millet was planted in raw soil at a distance of about 10 cm between plants and a distance between rows of about 30 cm. During the experiment, the average daily temperatures ranged from 23.2 °C in June to 15.7 °C in October 2016. The average relative humidity was 75.2%. The soil texture of the field was classified as loam composed of sand (40.9%), silt (36.4%), and clay (22.7%) on the soil taxonomy of the United States Department of Agriculture^21^. The moisture content of the soil was 10.7% and the pH value was 5.0. The organic matter was 1.72% and the cation exchange capacity was 8.0 meq/100 g.

### Pesticide application

**Figure 2:**
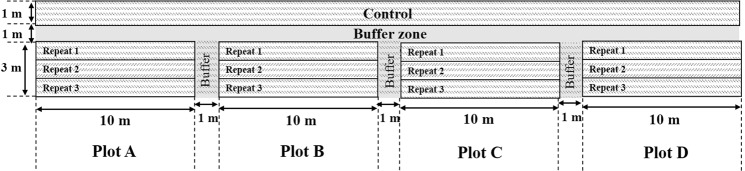
Experimental design of the plots for the field experiment.

The experimental field was divided into four plots according to pesticide treatment dates. Each plot was 30 m^2^ and contained three replicates of 10 m^2^. Three sections of each plot were treated with the pesticide twice as follows: Plot A was sprayed 14 and 7 days before harvest, plot B was sprayed 21 and 14 days before harvest, plot C was sprayed 30 and 21 days before harvest, and plot D was sprayed 40 and 30 days before harvest. The buffer zones were installed to separate each plot to prevent cross-contamination within different treatment plots. The arrangement of the field experiment is illustrated in Fig. 2. The cyantraniliprole OD formulation was 1,000 times with water, and sprayed until the grain and straw were saturated using a pressurized handgun-sprayer (20 L) in accordance with general farming custom. Before pesticide treatments, the reproducibility of the handgun sprayers was tested to ensure that the treatment remained consistent with regard to spraying capacity and speed.

### Crop sampling

Seven days from the final treatment for plot D (14/7 days), both grain and straw samples were randomly harvested to a weight of 1 kg. The straw samples were cut to the size of 3~5 cm before homogenization. The grain was air-dried in darkness for 2 days and it was manually threshed. The prepared grain and straw were macerated together with dry ice using a blender (Hanil, HMF-3100S, Republic of Korea). The fine powder samples were placed into a polyethylene bag and kept in a freezer at −20 °C.

### Sample preparation

The citrate-buffered QuEChERS method, which is the standardized method recommended by the European Committee^22^, was employed for the extraction of cyantraniliprole and IN-J9Z38. The detailed procedure was as follows: 5.0 ± 0.1 g of homogenized proso millet grain and straw was weighed in a 50-mL centrifuge tube. After adding 5 mL of deionized water, the tube was vortexed and left to soak in the water for 30 minutes. Ten milliliters of acetonitrile was added to the tube with a ceramic homogenizer, followed by vigorous shaking using a mechanical shaker (MMV-1000W, EYELA, Japan) for 30 minutes. Then, the tubes were stored in an ice bath in order to prevent the heat generation caused by magnesium sulfate absorbing water. Subsequently, the QuEChERS extraction package containing 4 g of magnesium sulfate, 1 g of sodium chloride, 1 g of trisodium citrate dihydrate, and 0.5 g of disodium hydrogen citrate sesquihydrate was added to each tube and shaken again for 1 minute. After centrifugation at 3,500 rpm for 5 minutes, the supernatant (1 mL) was transferred to a dSPE clean-up tube containing 25 mg of PSA and 150 mg of magnesium sulfate. The tubes were vigorously vortexed for 1 minute and centrifuged at 13,000 rpm for 5 minutes. Finally, the upper layer extract was diluted with acetonitrile by the ratio of 1:1 prior to injection into the UHPLC-MS/MS.

### Instrumental conditions

Analysis of cyantraniliprole and IN-J9Z38 was performed on a LCMS-8040 (Shimadzu, Japan) coupled with Nexera UHPLC (Shimadzu, Japan). Positive mode in electro-spray ionization (ESI) was used for the MS/MS detection with the following MS parameters: the capillary voltage was 4.0 kV, and nitrogen was the nebulizing gas (3.0 L/min) and drying gas 15.0 L/min). The desolvation line temperature was 250 °C and the heat-block temperature was 400 °C. Chromatographic separation for UHPLC was carried out on a Kinetex C18 column (100 mm × 2.1 mm, 2.6 μm, Phenomenex, CA, USA) at a 40 °C column temperature. The mobile phase consisted of deionized water (A) and methanol (B), both containing 0.1% formic acid and 5 mM ammonium formate. The flow rate of the mobile phase was 0.2 mL/min and the gradient was programmed as follows: initially, 5% of the organic solvent mobile phase (B) was kept constant for 1 min followed by increasing it to 95% (B) linearly over 2.5 min and held for 3.5 min. Finally, the ratio of B was restored to 5% over 0.5 min and maintained for 2.5 min. The total analytical running time was 10 min and the injection volume was 5 μL. Highly selective MS/MS detection was achieved by multiple reaction monitoring (MRM). The MRM transition pair of quantitation and identification was optimized by direct injection of standard solution (1 μg/mL) without an analytical column.

### Method validation

The analytical method for proso millet grain and straw was validated in terms of the instrumental limit of quantitation (ILOQ), method limit of quantitation (MLOQ), linearity of the calibration curve, and recovery. The lowest concentration where the signal-to-noise ratio was higher than 10 was defined as ILOQ. MLOQ was calculated by multiplying the ILOD by four, which was the dilution factor of the sample preparation method. The recovery test was carried out to evaluate accuracy and precision by fortifying a pesticide standard solution. Five grams of control grain and straw samples were spiked with the pesticides mixture solution to make the concentrations of 0.1 and 0.5 mg/kg (*n* = *3*). The fortified samples and control samples were analyzed with the method mentioned above, and then recovery rates (%) were calculated by comparing the detected concentrations with the fortified concentrations. The precision was also evaluated by relative standard deviation (RSD, %) of recoveries within replicates. The linearity of the calibration curve in each matrix and compound was evaluated using seven points (0.0025, 0.005, 0.010, 0.025, 0.05, 0.1, and 0.25 μg/mL) of matrix-matched standard, which was prepared by dilution with control extracts at a ratio of 1:1.

### Storage stability test

The stability of cyantraniliprole residue under sample storage conditions was tested. Grain and straw samples for both fortified (*n* = *3*) and field samples were kept in the same freezer for 25 days (−20 °C in darkness). The fortified samples were spiked with cyantraniliprole and IN-J9Z38 at a level of 0.5 mg/kg (50 times of MLOQ). Storage for both of the samples was initiated at the same time, and they were analyzed together on the same day of sample analysis. Stability was expressed by the recovery rate of cyantraniliprole and IN-Z9Z38 residue in the sample.

### Calculation of cyantraniliprole residue pattern

The residual concentrations in the field samples were expressed as the sum of the parent compound (cyantraniliprole) and its metabolite (IN-J9Z38) by the following equation, where the constant of 1.04 is the conversion factor calculated by dividing the molecular weight of cyantraniliprole (473.2 *m/z*) by the molecular weight of IN-J9Z38 (455.7 *m/z*):

$$\begin{array}{ccl}{\rm{T}}{\rm{o}}{\rm{t}}{\rm{a}}{\rm{l}}\,{\rm{r}}{\rm{e}}{\rm{s}}{\rm{i}}{\rm{d}}{\rm{u}}{\rm{e}}{\rm{s}}\,{\rm{o}}{\rm{f}}\,{\rm{c}}{\rm{y}}{\rm{a}}{\rm{n}}{\rm{t}}{\rm{r}}{\rm{a}}{\rm{n}}{\rm{i}}{\rm{l}}{\rm{i}}{\rm{p}}{\rm{r}}{\rm{o}}{\rm{l}}{\rm{e}}({\rm{m}}{\rm{g}}/{\rm{k}}{\rm{g}}) & = & {\rm{C}}{\rm{y}}{\rm{a}}{\rm{n}}{\rm{t}}{\rm{r}}{\rm{a}}{\rm{n}}{\rm{i}}{\rm{l}}{\rm{i}}{\rm{p}}{\rm{r}}{\rm{o}}{\rm{l}}{\rm{e}}\,{\rm{r}}{\rm{e}}{\rm{s}}{\rm{i}}{\rm{d}}{\rm{u}}{\rm{e}}\,({\rm{m}}{\rm{g}}/{\rm{k}}{\rm{g}})\\  &  & +[1.04\times {\rm{M}}{\rm{e}}{\rm{t}}{\rm{a}}{\rm{b}}{\rm{o}}{\rm{l}}{\rm{i}}{\rm{t}}{\rm{e}}\,{\rm{r}}{\rm{e}}{\rm{s}}{\rm{i}}{\rm{d}}{\rm{u}}{\rm{e}}\,({\rm{m}}{\rm{g}}/{\rm{k}}{\rm{g}})].\end{array}$$

The dissipation pattern in grain and straw was expressed by the function of exponential decay as follows: C_*t*_ = C_0_ × e^*−kt*^, where *t* is the number of days after pesticide treatment, C_0_ is the highest concentration of total cyantraniliprole residue, and *k* is the dissipation rate constant. In accordance with the equation obtained from the field data, the biological half-life in days (DT_50_) was calculated from the following equation: DT_50_ = ln (0.5)/*k*^23–26^.

## Results and Discussion

### Optimization of UHPLC-MS/MS detection

**Figure 3:**
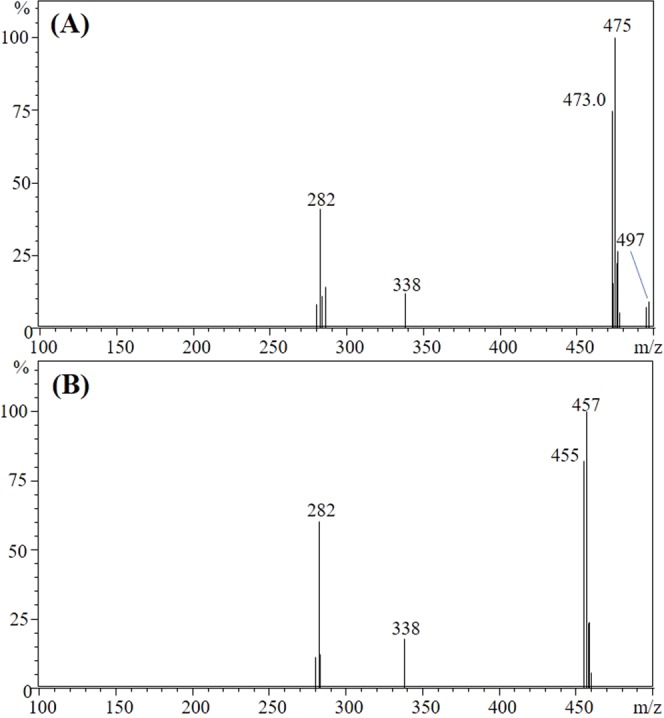
UHPLC-MS/MS full-scan spectra of cyantraniliprole (**A**) and IN-J9Z38 (**B**) obtained by direct injection of standard solutions (1.0 μg/mL).

A number of parameters in the ESI module are involved in ionization of the target compound in LC-MS/MS. Therefore, MRM conditions in LC-MS/MS may vary depending on instrumental conditions such as mobile phase, nebulizing/desolvation gas, and variable voltage conditions^27^. In this study, the MRM parameters were adjusted for the sensitive and selective detection of target compounds. For selection of the precursor ion, the full scan spectrums of cyantraniliprole and IN-J9Z38 were obtained in the range of 100–500 *m/z* by direct injection with the flow of the mobile phase (50:50 as a mobile phase ratio of A and B). The spectrums obtained are depicted in Fig. 3. Cyantraniliprole (the monoisotopic mass is 472 Da) produced 475 *m/z* as a base ion with the form of the protonated isotopic molecular ion [M + 2 + H]^+^. The protonated molecular ion ([M + H]^+^) of 473 *m/z* was also observed with high intensity on the spectrum, but it was relatively lower than the base ion (475 *m/z*). Interestingly, the ratio between [M + 2 + H]^+^ and [M + H]^+^ matched the theoretical value (4:3) that was produced by one chlorine and one bromine atom in cyantraniliprole. The specific isotopic pattern of a chlorine atom (3:1 isotope ratio for ^35^Cl and ^37^Cl) and a bromine atom (1:1 ratio for ^79^Br and ^81^Br) resulted in the unusual isotopic ratio in the spectrum. In addition, the sodium adducted molecular ion [M + 2 + Na]^+^ of 497 *m/z* was also found, but the intensity we found was significantly lower than the results from a previous study that gave the highest intensity^15^. Similarly, the full scan spectrum of IN-J9Z38 (the exact mass is 454 *m/z*) also had the high intensity of 457 *m/z* ion from [M + 2 + H]^+^ as the base ion. The 455 *m/z* of [M + H]^+^ was also observed with the same ion ratio (4:3) as cyantraniliprole (Fig. 3B).

The base ion in both cyantraniliprole (475 *m/z*) and IN-J9Z38 (457 *m/z*) was selected as a precursor ion to produce the product ion. From the selected precursor ions, the product scan spectrum was obtained using various collision energies (0–50 eV). The two transitions that showed the best sensitivity and selectivity, including the product ions and collision energies, were chosen for quantitation and qualification transition. The relative abundance values (quantitation ion/qualification ion) within two transitions were 34% and 78% for cyantraniliprole and IN-J9Z38, respectively. The ion ratio tolerance of 30% was maintained for all sample analyses to avoid false-positive or -negative quantitation.

Due to a large number of field sample experiments, faster chromatographic separation was performed to reduce analysis time. To achieve the best separation in the mobile phase, methanol and water were chosen for use with formic acid and ammonium formate. Not only did it provide a shorter run time, it also resulted in a desirable peak shape when using the Kinetex C18 column with 2.6-μm particle size. Details of the MRM transitions and retention times are presented in Table 1.

**Table 1:** Retention times and multiple reaction monitoring (MRM) transitions used for UHPLC-MS/MS analysis.

Compound	t_R_^a^ (min)	Monoisotopic mass (Da)	Ionization	Precursor ion > Product ion (CE^b^, voltage)	
				Quantitation transition	Qualification transition
Cyantraniliprole	4.68	472.0	[M + 2 + H]^+^	475.0 < 285.9 (−14)	475.0 < 443.9 (−16)
IN-J9Z38	4.97	454.0		457.0 < 112.0 (−53)	457.0 < 299.0 (−39)

^a^*t*_*R*_ Retention time, ^b^*CE* Collision energy.

### Method validation

Since it was first introduced in 2003 by Anastassiades *et al*.^28^, QuEChERS methodology has been widely used by many researchers for the analysis of pesticides. Two versions (the so-called original and AOAC QuEChERS) of the QuEChERS approach has been reported for the analysis of cyantraniliprole and IN-J9Z38^15,29^. These two versions could have been used in this study; however, we employed simple and rapid analysis of cyantraniliprole and IN-J9Z38 by the EN 15662 method. The results of this citrate-buffered version of the QuEChERS approach confirmed that various types of QuEChERS approaches are generally applicable to the analysis of cyantraniliprole and IN-J9Z38 in various matrices.

LOQ and MLOQ are important parameters to measure the sensitivity of an instrument or analytical method in method validation. Among the many different ways to present an ILOQ, the minimum concentration that provided a signal to noise ratio over 10 was defined as an ILOQ in this study^30,31^. The cyantraniliprole and IN-J9Z38 had a low ILOQ of 0.0025 μg/mL in the matrix-matched standard solution in both the grain and straw. The MLOQ was set at 0.01 mg/kg by calculating the dilution factor of the sample preparation method and matrix matching procedure. This MLOQ was regarded as suitable to quantitate the field samples in consideration of the lowest MRL registered in cyantraniliprole (0.01 mg/kg, Korean MRL).

The linearity of the calibration curve including the equation and regression coefficients is presented in Table 2. Satisfactory linearity was obtained with coefficient values of determination (*r*^2^) greater than 0.998 in all cases. However, a small difference was observed in the slope of the calibration curve within grain and straw, even in the same compound. This result indicates that the signal response can differ from matrices and requires compensation by matrix-matched calibration for reliable quantitation.

**Table 2:** Linearity values (*r*^2^), equations of the calibration curve, and recovery test results in proso millet grain and straw.

Compound	Matrix	Spiked levels (mg/kg)	Average recovery (%) ± RSD^a^ (%)	Equation of calibration curve	Regression coefficient (*r*^2^)
Cyantraniliprole	Grain	0.1	91.2 ± 5.5	*y* = 1240370.2*x* + 558.0	0.9995
		0.5	92.1 ± 1.4		
	Straw	0.1	105.3 ± 2.0	*y* = 1062630.9*x* −1691.7	0.9990
		0.5	101.0 ± 0.9		
IN-J9Z38	Grain	0.1	88.8 ± 5.5	*y* = 139138.3*x* −53.9	0.9995
		0.5	91.7 ± 5.9		
	Straw	0.1	108.2 ± 2.5	*y* = 111534.5*x* −211.5	0.9983
		0.5	102.5 ± 4.6		

^a^*RSD* Relative standard deviation.

**Figure 4:**
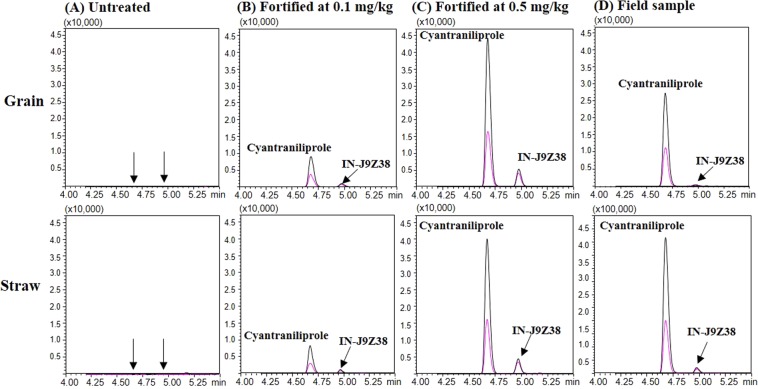
Representative UHPLC-MS/MS chromatograms of untreated (**A**), recovery samples fortified at 0.1 and 0.5 mg/kg (**B**,**C**), and field samples sprayed 7–14 days before harvest (**D**) for proso millet grain and straw

Accuracy and precision were also evaluated by the recovery rate (%) and RSD (%) value from the recovery tests (Table 2). Overall, good recovery rates of 91.2–105.3% for cyantraniliprole and 88.8–108.2% for IN-J9Z38 were obtained across all spiked levels (0.1 and 0.5 mg/kg) and matrices (grain and straw). The precision, expressed as RSD, was less than 5.9% for all of the results. Figure 4 shows the representative chromatograms of cyantraniliprole and IN-J9Z38 in the recovery tests and samples from field experiments. These results showed no interference from co-eluted peaks around the retention times in both grain and straw samples (Fig. 4). Thus, the satisfactory results from the recovery test demonstrated that the analytical method could be applied to quantitation of cyantraniliprole and IN-J9Z38 residues in the field samples.

### Stability testing during sample storage

Due to the large number of samples collected, storage was required prior to analysis. Therefore, storage testing was performed to confirm the stability of target compounds under storage conditions. Stability was evaluated as a recovery rate (%) representing the relative ratio between the determined concentration and the spiked concentration (0.5 mg/kg). The recovery rate ranged from 91.9% to 103.7% for cyantraniliprole and 90.8% to 109.0% for IN-J9Z38. Although the spiked samples were stored for 25 days, there were no significant differences in the spiked concentration on testing.

### Dissipation pattern of cyantraniliprole in proso millet

The analytical method of cyantraniliprole was successfully applied to field proso millet samples. No residue was detected in the control sample from the untreated plot. The residue amounts of cyantraniliprole and IN-J9Z38 in the field experiment are shown in Table 3. As the duration from last pesticide application to harvest date increased, cyantraniliprole residue levels decreased steadily with time. Cyantraniliprole residues in grain and straw showed a decline between plot A (7 days before harvest) and plot D (30 days before harvest) of 91.1% and 89.1%, respectively. Cyantraniliprole residues in grain ranged from 0.03 to 0.33 mg/kg, relatively higher than those in a previously reported study of rice. Lower residues (≤0.04 mg/kg) were found in rice despite treatment with similar amounts of the active ingredient^19^. On the other hand, residue levels of the metabolite IN-J9Z38 gradually increased with duration leading up to harvest. While residue levels were not high, the 0.02 mg/kg IN-J9Z38 residue increased to 0.06 mg/kg over 30 days. These data suggest that cyantraniliprole may be metabolized or transformed into IN-J9Z38 during cultivation. Our observation of this metabolite sparks interest because other studies have reported non-detectable or trace levels of IN-J9Z38 residues in Chinese cabbage, rice, tomatoes, cucumbers, and soil^15,19,20,29^.

**Table 3:** Dissipation patterns of cyantraniliprole and IN-J9Z38 in proso millet.

Plot	Pesticide treatment^a^	Grain				Straw			
		Cyantraniliprole		IN-J9Z38		Cyantraniliprole		IN-J9Z38	
		Residue^*b*^	Dissipation, %	Residue	Dissipation, %	Residue	Dissipation, %	Residue	Dissipation, %
Plot A	14-7	0.33 ± 0.11	—	0.02 ± 0.01	—	0.98 ± 0.32	—	0.35 ± 0.16	—
Plot B	21–14	0.23 ± 0.03	31.0	0.04 ± 0.01	—	0.30 ± 0.09	69.3	0.16 ± 0.04	54.3
Plot C	31-21	0.17 ± 0.06	47.6	0.07 ± 0.03	—	0.17 ± 0.02	82.9	0.12 ± 0.02	64.8
Plot D	40-30	0.03 ± 0.02	91.1	0.06 ± 0.06	—	0.11 ± 0.01	89.1	0.12 ± 0	65.7

^a^Pesticide treatment: days before harvest, ^*b*^Residue: mg/kg, average residue ± standard deviation (*n* = 3).

In straw, a similar dissipation pattern was observed for cyantraniliprole, but relatively higher residues were observed than for grain. Cyantraniliprole residues decreased from 0.98 mg/kg in plot A to 0.11 mg/kg in plot D, an 89.1% dissipation rate. IN-J9Z38 residue in straw also decreased from 0.35 to 0.12 mg/kg, unlike in grain. Although the MRLs of pesticides apply only to edible parts, straw analysis was also conducted to determine whether pesticide was properly sprayed with a reasonable residue level. In addition, determining differences in dissipation patterns between grain and straw can be useful when estimating pre-harvest intervals for safe use of pesticides during cultivation^32^.

**Figure 5:**
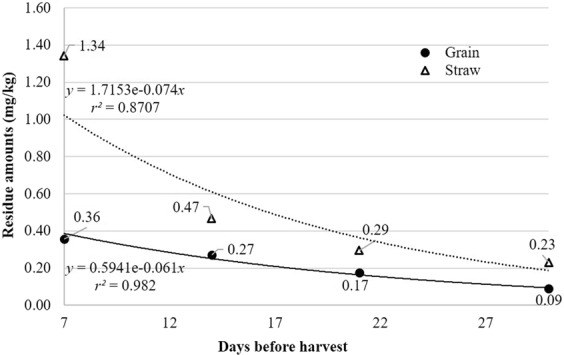
Dissipation patterns for total cyantraniliprole residue in proso millet grain and straw.

Figure 5 shows the curve for total cyantraniliprole, which is expressed by the sum of cyantraniliprole and IN-J9Z38 residues in proso millet and straw. The equations for dissipation were calculated as C = 0.5941e^−0061t^ (grain) and C = 1.7153e^−0.0738t^ (straw) with the correlation coefficients (*r*^2^) of 0.9820 and 0.8707 for grain and straw, respectively. The DT_50_ values were 11.3 days (grain) and 9.4 days (straw), respectively, which were relatively longer than those in other crops: 3.2–6.3 days in brown rice and straw^19^, 2.2 days in cucumbers, and 2.8 days in tomatoes^23^. These studies, including ours, used the same pesticide formulation (OD) and active ingredient content (10%). This suggests that the dissipation and half-life of pesticide residues can vary considerably based on other factors, such as field environment (e.g., temperature, humidity, sunlight, and rainfall), type of plant (e.g., species, structure, permeability, and growth rate), metabolic activity, and many other variables^33–36^. In addition, no growth of proso millet was observed in the period of pesticide application. The dilution effect caused by growth of a crop has been reported to be a major factor that contributes to the decreased half-lives of pesticides, as seen in cucumbers and tomatoes^37–39^. In our experiment, this phenomenon is negligible due to the lack of growth following pesticide application.

In conclusion, simple and simultaneous determination of cyantraniliprole and its metabolite residues in proso millet was achieved using the QuEChERS method and UHPLC-MS/MS. The analytical method was validated in terms of ILOQ, MLOQ, linearity of the calibration curve, accuracy, and precision. The validated analytical method was successfully applied to field samples. The dissipation study showed that the half-lives of cyantraniliprole were 11.3 and 9.4 days for grain and straw, respectively, and these results were compared with those of previous studies. The results of this study will inform the regulation and management of pesticide use for the minor crop proso millet.
